# Standard RUTF vs. locally-made RUSF for acutely malnourished children: A quasi-experimental comparison of the impact on growth and compliance in a rural community of Pakistan

**DOI:** 10.1371/journal.pone.0287962

**Published:** 2023-07-12

**Authors:** Azza Sarfraz, Sheraz Ahmed, Sajid Muhammad, Najeeb Rehman, Sanam Iram Soomro, Khaliq Qureshi, Sadaf Jakhro, Fayaz Umrani, Adam Greene, Sana Syed, Sean R. Moore, Syed Asad Ali

**Affiliations:** 1 Department of Pediatrics and Child Health, Aga Khan University, Karachi, Pakistan; 2 Department of Pediatrics, University of Virginia, Charlottesville, VA, United States of America; Public Library of Science, UNITED KINGDOM

## Abstract

**Background:**

The reduction in severe and moderate acute malnutrition (SAM and MAM) rates in Pakistan has been sub-optimal compared to other low-and middle-income countries (LMICs). Specially-formulated products have been designed globally to manage SAM and MAM, such as ready-to-use therapeutic food (RUTF) and ready-to-use supplementary food (RUSF), with variable efficacies. RUTF is primarily produced and patented in industrialized countries, raising supply challenges in resource-constrained regions with a high burden of acute malnutrition. RUSF minimizes costs by using locally-available ingredients while providing similar nutritional value. In this study, we compared the efficacy, side effects, and compliance of two months of supplementation with either RUTF or RUSF.

**Methods:**

Children aged nine months in the rural district of Matiari, Pakistan, with a weight-for-height z-score (WHZ) <-2 received either RUTF (500 kcal sachet) for two months in 2015 or RUSF (520 kcal sachet) for two months in 2018.

**Results:**

The RUSF group had a higher height gain and mid-upper arm circumferences (MUAC) score. Higher compliance was noted with lower side effects in the RUSF group. A higher compliance rate did correlate with the growth parameters in respective groups.

**Conclusion:**

Our study found that both RUTF and RUSF partially improve the anthropometric status of acutely malnourished children, with neither being superior to the other.

## 1. Background

Stunting, wasting, and underweight are widely recognized forms of malnutrition. Wasting is an acute form of malnutrition in which a child has low weight-for-height, classified as either moderate acute malnutrition (MAM) or severe acute malnutrition (SAM). Mid-upper arm circumference (MUAC) is another anthropometric measure used to predict malnutrition-associated outcomes including SAM (<115 mm) and MAM (≥115 mm and <125 mm). SAM is particularly life-threatening and requires early detection and treatment before the onset of complications. Per the latest World Health Organization (WHO) estimates, 51 million children under the age of five are wasted, of whom 17 million are severely wasted. Recently, there has been a rise in wasting or acute malnutrition prevalence in Pakistan, increasing from 15.1% in 2011 to 17.7% in 2018 [1]. Many products and approaches have been considered in the past two decades to prevent and treat acute malnutrition in children [2, 3], including ready-to-use therapeutic food (RUTF), a lipid-based nutrient supplement (such as peanut-based Plumpy’Nut®). WHO/UNICEF protocols have endorsed RUTF for treating uncomplicated SAM due to their relatively higher energy content, longer shelf life, and overall efficacy [4]. However, RUTF is costly, primarily produced and patented in high-income countries (HICs) [5]. The centralized production of RUTF in industrialized countries results in supply chain challenges to regions with a higher burden of malnutrition, such as Pakistan, which has been further delayed since the COVID-19 pandemic.

**Fig 1:**
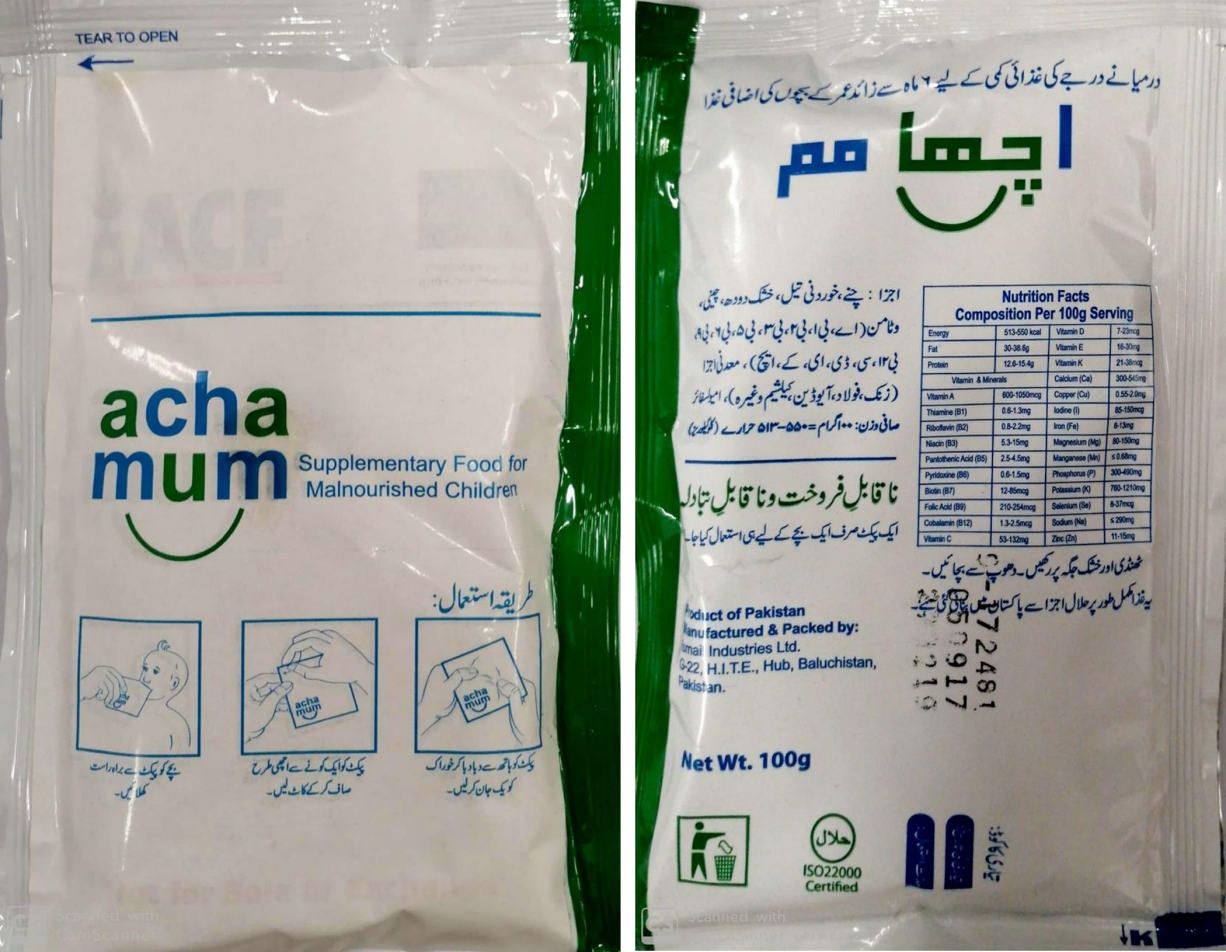
**a.** Front side of AchaMum ready-to-use supplementary food (RUSF). **b.** Back side of AchaMum ready-to-use supplementary food (RUSF).

These challenges stimulated the development of locally-made fortified food blends, known as ready-to-use supplementary food (RUSF). RUSF provides higher energy for children with moderate acute malnutrition (MAM) and is more cost-effective, socially acceptable, and readily available [6, 7]. Currently, there is no standardized nutrient composition of RUSF to manage acute malnutrition [8]. Several RUSFs have been formulated and tested across malnourished endemic regions such as Africa and South Asia, demonstrating variable efficacy [7, 9–11]. Current treatments also differentiate SAM from MAM by prescribing different products and protocols [12]. A combined protocol for SAM and MAM with RUSF developed locally may prove more therapeutically- and cost-effective in children under five across low- and middle-income countries (LMICs) with a high burden of malnutrition [13]. Even though many guidelines propose either the use of RUSF or RUTF for different clinical syndromes, in terms of nutritional composition, they are essentially the same as shown in **Fig 1**. In this study, we compared the growth trends, compliance, and side effects of a well-recognized RUTF (Plumpy’Nut®) against a locally-made RUSF (AchaMum) in a rural population of moderate-to-severely malnourished children.

## 2. Methodology

### 2.1 Description of cohorts and study site

The Environmental Enteropathy Study (EES), the RUTF cohort, was conducted from April 2013 to November 2015. The Study of Environmental Enteropathy (SEEM), the RUSF cohort, was conducted from March 2016 to March 2019. The primary objective of both studies was to determine the biomarkers for environmental enteropathy in malnourished children. As we had experienced import challenges in administering RUTF (Plumpy’Nut®) in our first EES study [14, 15], we procured locally-made RUSF (AchaMum) for the subsequent SEEM study. We provided outpatient nutritional rehabilitation to uncomplicated SAM and MAM children, defined as weight-for-height z-score (WHZ) < -3 and WHZ < -2 and > -3, respectively. In EES, we treated SAM and MAM children with RUTF (n = 65), whereas in SEEM, we treated SAM and MAM children with locally-made RUSF (n = 187). Both studies were conducted in the rural district of Matiari, located in the Sindh province of Pakistan. The Department of Pediatrics and Child Health at Aga Khan University has a well-established research center in Matiari, one of Sindh province’s rural districts [14–21].

### 2.2 Study design

In both EES and SEEM, children were registered within one month of their birth and followed weekly until 18 months and 24 months old, respectively. On every weekly visit, project-hired data collectors recorded the number of days with reported symptoms of acute respiratory illness, vomiting, and diarrhea in the preceding week. Data collectors took monthly anthropometric measurements, including height (1 mm precision using a rigid length board with a movable foot piece) and weight (20 g precision electronic scale; TANITA 1584) of all enrolled children. In EES, we provided children identified as having moderate to severe malnutrition (WHZ <-2) at nine months of age with educational and nutritional rehabilitation interventions. The nutritional intervention was weight-appropriate RUTF for two months (200 kcal/kg/day), and data collectors monitored compliance weekly. In SEEM, from 3–6 months, if the child’s weight-for-height z-score (WHZ) was <-2, the child was enrolled by the study physician on revalidation of the score recorded by the data collectors. From 6 months to 9 months, parents of enrolled children were counseled on weaning and complimentary food supplementation through a ten-minute video message and one-on-one counseling every month. Children with a WHZ score of <-2 at nine months of age were eligible for nutrition intervention with weekly compliance monitoring. We provided RUSF intervention (one sachet per day for two months) to the children with WHZ <-2 but ≥-3. For WHZ <-3, the sachet was supplied as per child weight (200 kcal/kg/day), similar to RUTF [22]. Compliance was calculated based on the empty wrappers returned by mothers. Side effects, such as diarrhea, vomiting, and abdominal pain, were noted separately alongside the overall morbidity patterns of the study participants based on the mothers’ perceptions and recall. RUSF was readily available locally and administered to one of the cohorts because of the supply challenges of RUTF.

### 2.3 Data collection

Project-hired data collectors who could speak, read and write the local language (Sindhi) were recruited for the study. Each union council was divided into two areas for logistics purposes. Data and laboratory samples were collected at the domiciliary level. Study physicians were hired for the enrollment and management of sick children. We collected detailed data on maternal and paternal education, employment, obstetric history, socioeconomic status, household food insecurity index, child’s clinical assessment, and illness history and treatment at the time of enrollment.

### 2.4 Data quality assurance

In both EES and SEEM, five percent of all field activities were monitored by project monitors. Weighing machines were calibrated with standardized weights daily. Length boards for children and height scales for parents were calibrated biweekly. Monitoring mechanisms included accompanied and independent visits. Monthly anthropometric readings were reviewed daily for outliers and were validated within 24–48 hours by monitors. Quarterly anthropometric standardization sessions were arranged for workers.

### 2.5 Data management

In EES, data were collected and stored as hard copies, which were reviewed daily by the data editor for logical checks and form completion. The data management unit (DMU) entered all forms in a computerized system daily. In SEEM, data were entered directly via an electronic tablet into a customized application in the Android operating system. The application was programmed using XML and Java. Initial data entry was stored locally in the tablet’s internal memory and was synchronized daily at the local server in the Matiari data management unit. Server data was backed-up daily at local and remote servers in Karachi.

### 2.6 Statistical analysis

Statistical analysis was performed on the two obtained data sets (EES and SEEM). Descriptive statistics were reported as frequency percent, mean/median with standard deviation, and interquartile range (IQR) as appropriate. Independent two-sample T-test and Mann Whitney U test were used to test the difference in the continuous measures between the RUSF group and RUTF group children while the chi-square test was used to test the difference in the categorical measures between the RUSF group and RUTF group. The anthropometric indicator status is presented at the start and two months after the intervention. All growth indicators were compared post-intervention based on compliance, categorized as compliance <75% and ≥75% per the percent of empty sachets returned by the mother/guardian. The rate of growth change per month in both cohorts was calculated from birth until one year at 3-monthly intervals. All statistical analyses were performed using Stata 17, and p-values <0.05 were considered statistically significant.

### 2.7 Ethics statement

The study protocols for EES and SEEM were approved by the Ethical Review Committee (ERC) of Aga Khan University in 2013 (Protocol 2446-Ped-ERC-13) and 2015 (Protocol 3836-Ped-ERC-15), respectively. After parents or legal guardians obtained written informed consent, children were enrolled in both studies. All human subject research ethics were followed following relevant guidelines and regulations during the entire duration of the studies.

## 3. Results

**Fig 2:**
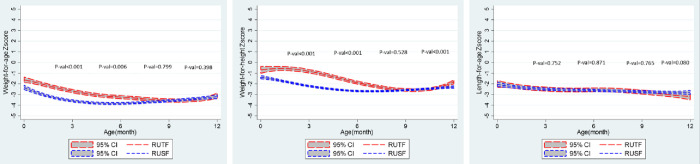
The trajectory of a) WAZ, b) WHZ, and c) HAZ from birth until 1 year of age in both cohorts. There is a significant difference in the WAZ and WHZ scores between RUTF and RUSF cohorts from 0–6 months.

The baseline characteristics of both cohorts at the time of enrollment are outlined in **Table 1**. A total of 65 participants in the RUTF group and 187 participants in the RUSF group were enrolled. 60% of children were born in the hospital in the RUTF group, compared to 76% in the RUSF group (p = 0.011). The average gestational age at birth was 36.6 weeks among the RUTF group, comparatively lower than the 38.5 weeks for the RUSF group (p<0.001). Notably, the maternal age was higher within the RUTF group, with 70% of mothers aged 30–49, compared to only 44% of mothers in the RUSF group (p<0.001). Both the groups significantly differed in WHZ and WAZ scores from registration until 6 months (p<0.001) but they were similar at the time of intervention at 9 months (**Fig 2**).

**Table 1:** Baseline characteristics of children at the time of registration.

Characteristics	RUTF group (n = 65)	RUSF group (n = 187)	P-values
**Age at time of registration (days)**	5.1 ± 4.1	6.6 ± 6.4	0.077
**Newborn gender**			0.220
Male, n (%)	35 (53.8)	117 (62.6)	
Female, n (%)	30 (46.2)	70 (37.4)	
**Birthplace**			0.011
Hospital/Maternity home, n (%)	39 (60.0)	142 (76.3)	
Home, n (%)	26 (40.0)	44 (23.7)	
**Gestational age at birth (weeks)**	36.6 ± 1.0	38.5 ± 1.3	<0.001
**Age of mother**			<0.001
15–29 years, n (%)	19 (29.7)	104 (55.6)	
30–49 years, n (%)	45 (70.3)	83 (44.4)	
**Mother education**			
No formal education, n (%)	58 (89.2)	166 (88.8)	0.920
Literate, n (%)	7 (10.8)	21 (11.2)	
**Child ever breastfed**			0.790
Yes, n (%)	64 (98.5)	177 (97.3)	
No, n (%)	1 (1.5)	4 (2.2)	
Don’t know, n (%)	0 (0.0)	1 (0.5)	
**Early initiation of breastfeeding**			0.024
Immediately after birth (first hour), n (%)	12 (18.8)	19 (10.5)	
1–6 hours after birth, n (%)	31 (48.4)	102 (56.4)	
7–12 hours after birth, n (%)	16 (25.0)	24 (13.3)	
13–24 hours after birth, n (%)	3 (4.7)	24 (13.3)	
More than 24 hours, n (%)	2 (3.1)	12 (6.6)	
Height for Age z-score*			0.970
<-3, n (%)	12 (18.8)	34 (18.4)	
≥-3 and <-2, n (%)	18 (28.1)	55 (29.7)	
≥-2, n (%)	34 (53.1)	96 (51.9)	
HAZ, median (IQR)	-1.8 (-2.7–1.2)	-1.9 (-2.7–1.2)	0.880
**Weight for height z-score** *			0.028
<-3, n (%)	0 (0.0)	11 (7.7)	
≥-3 and <-2, n (%)	6 (12.0)	31 (21.7)	
≥-2, n (%)	44 (88.0)	101 (70.6)	
WHZ, median (IQR)	-0.4 (-1.2–0.2)	-1.46 (-2.1–0.7)	<0.001
**Weight for Age z-score** *			0.012
<-3, n (%)	8 (12.3)	46 (24.9)	
≥-3 and <-2, n (%)	17 (26.2)	63 (34.1)	
≥-2, n (%)	40 (61.5)	76 (41.1)	
WAZ, median (IQR)	-1.55 (-2.5–1.1)	-2.2 (-3.0–1.6)	<0.001

* Within one week of the age of birth

**Fig 3:**
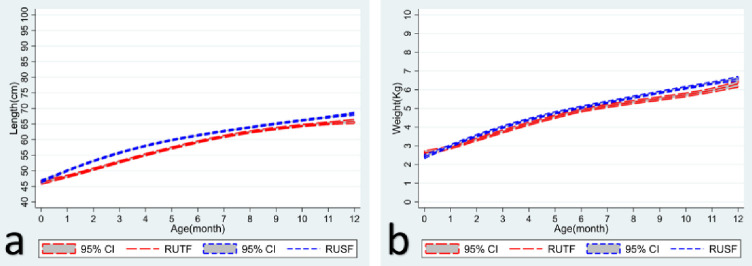
The trajectory of a) weight (kgs) and b) height (cms) from birth until 1 year of age in both cohorts.

The anthropometric indicators are outlined in **Table 2**, and growth trajectories until one year for both cohorts are shown in **Figs 2 and 3**. The average increase in weight during the two months of intervention in the RUTF group was 620 grams compared to the 680 grams in the RUSF group (p>0.290). During the same period, the average height increase was 1.4 cm in the RUTF group and 2.2 cm in the RUSF group (p<0.001). With MUAC as the anthropometric indicator, the proportional change in MUAC from <115mm to >115 mm at the end of the intervention was lower in the RUTF group compared to the RUSF group (31% vs. 42%, p<0.001).

**Table 2:** Anthropometric indicators of children at the time of intervention (9 months), one month, and two months after the intervention.

	RUTF group (n = 65)	RUSF group (n = 187)	P-values
**Age (months) at the time of initiation of intervention, Mean ± SD**	9.0 ± 0.2	9.9 ± 1.5	<0.001
**Height-for-Age**			
HAZ <-2 at the time of initiation of intervention, n (%)	45 (75.0)	100 (68.0)	0.320
HAZ <-2 at One month after intervention, n (%)	50 (82.0)	108 (75.0)	0.280
HAZ <-2 at Two months after intervention, n (%)	50 (82.0)	92 (73.6)	0.210
Δ*HAZ, (Mean ± SD)	-0.25 ± 0.2	-0.05 ± 0.4	<0.001
Δ*Height, (Mean ± SD), (centimeters)	1.38 ± 0.6	2.16 ± 0.9	<0.001
**Weight-for-Height**			
Δ*WHZ during two months of the intervention (Mean ± SD)	0.83 ± 0.6	0.41 ± 0.9	<0.001
WHZ < -3 at the time of initiation of intervention, n (%)	16 (25.4)	36 (24.5)	0.890
WHZ < -2 to -3 at the time of initiation of intervention, n (%) **	45 (71.4)	94 (63.9)	0.290
WHZ < -3 at one month after intervention, n (%)	14 (21.5)	32 (22.1)	0.930
WHZ < -2 to -3 at one month after intervention, n (%)	30 (46.2)	72 (49.7)	0.640
WHZ < -3 at two months after intervention, n (%)	4 (6.2)	28 (21.9)	0.006
WHZ < -2 to -3 at two months after intervention, n (%)	17 (26.2)	48 (37.5)	0.110
**Weight-for-Age**			
Δ*Weight, Mean ± SD (grams)	620 ± 300	680 ± 500	0.290
Δ*WAZ, Mean ± SD	0.50 ± 0.4	0.36 ± 0.6	0.110
WAZ < -2 at the time of initiation of intervention, n (%)	60 (98.4)	135 (95.7)	0.350
WAZ < -2 at one month after intervention, n (%)	62 (98.4)	126 (90.6)	0.044
WAZ < -2 at two months after intervention, n (%)	57 (89.1)	112 (90.3)	0.790
**MUAC**			
<115 mm at the start of intervention, n (%)	36 (56.3)	38 (25.5)	<0.001
115mm-124mm at the start of intervention, n (%)	23 (35.9)	55 (36.9)	
≥125mm at the start of intervention, n (%)	5 (7.8)	56 (37.6)	
<115 mm at one month after intervention, n (%)	32 (49.2)	26 (17.8)	<0.001
115mm-124mm at one month after intervention (n, %)	29 (44.6)	52 (35.6)	
≥125mm at one month after intervention, n (%)	4 (6.2)	68 (46.6)	
<115 mm at two months after intervention, n (%)	25 (38.5)	22 (17.1)	<0.001
115mm-124mm at two months after intervention, n (%)	31 (47.7)	43 (33.3)	
≥125mm at two months after intervention, n (%)	9 (13.8)	64 (49.6)	

* Δ = Change during the two months of intervention

**Some children at borderline WHZ such as -1.9 who had a declining growth trend were also given nutritional intervention despite not being at the anthropometric cut-off.

When observing compliance across both groups, the RUTF group had lower average compliance than the RUSF group, with mean two-month compliance of 45%, compared to 81% for RUSF (p<0.001) (**Table 3**).

**Table 3:** Compliance across groups in the 1^st^ and 2^nd^ months of intervention.

	RUTF group (n = 65)	RUSF group (n = 187)	
Compliance* at one month after intervention**			
<75% compliance, n (%)	48 (90.6)	74 (40.2)	<0.001
≥75% compliance, n (%)	5 (9.4)	110 (59.8)	
Average compliance, n (%)	37.2 ± 25.3	76.5 ± 23.4	<0.001
Compliance* at two months after the intervention**			<0.001
<75% compliance, n (%)	44 (83.0)	52 (29.5)	
≥75% compliance, n (%)	9 (17.0)	124 (70.5)	
Average compliance, n (%)	45.1 ± 26.3	81.3 ± 24.0	<0.001

* Compliance = [(Total packet used/total packet given) *100]

** Some participants were either lost to follow-up or the visit was not conducted in that time frame.

Limiting our analysis to the compliance of nutritional intervention (<75% and ≥75%) with anthropometric indicators at 2 months after initiation, we reported key findings in **Table 4**.

**Table 4:** Compliance-based association of anthropometric indicators among children at 1 and 2 months after initiation of intervention.

	After two months					
Compliance	Compliance <75%			Compliance ≥75%		
Group	RUTF group (n = 44)	RUSF group (n = 52)	P-values	RUTF group (n = 9)	RUSF group (n = 124)	P-values
**Height-for-Age**						
Δ*Height (centimeters) during two months of the intervention, Mean ± SD	1.55 ± 0.61	2.19 ± 0.99	<0.001	1.29 ± 0.71	2.14 ± 0.81	0.003
Δ*HAZ, Mean ± SD	-0.24 ± 0.23	-0.04 ± 0.38	0.008	-0.26 ± 0.31	-0.05 ± 0.34	0.110
<-3, n (%)	15 (35)	18 (55)	0.026	4 (50.0)	43 (46.7)	0.700
≥-3 and <-2, n (%)	19 (44)	5 (15)		3 (37.5)	26 (28.3)	
≥-2, n (%)	9 (21)	10 (30)		1 (12.5)	23 (25.0)	
**Weight-for-Height**						
Δ*WHZ during two months of the intervention, Mean ± SD	0.84 ± 0.60	0.13 ± 0.75	<0.001	0.76 ± 0.41	0.52 ± 0.86	0.420
<-3, n (%)	2 (5)	10 (29)	<0.001	1 (11.1)	18 (19.4)	0.500
≥-3 and <-2, n (%)	10 (23)	16 (46)		2 (22.2)	32 (34.4)	
≥-2, n (%)	32 (73)	9 (26)		6 (66.7)	43 (46.2)	
**Weight-for-Age**						
Δ*Weight (grams), Mean ± SD	740 ± 360	490 ± 400	0.005	600 ± 230	670 ± 450	0.630
Δ*WAZ, Mean ± SD	0.53 ± 0.42	0.23 ± 0.59	0.014	0.44 ± 0.28	0.40 ± 0.64	0.890
<-3, n (%)	16 (36)	17 (53)	0.340	5 (62.5)	49 (53.3)	0.640
≥-3 and <-2, n (%)	22 (50)	12 (38)		3 (37.5)	34 (37.0)	
≥-2, n (%)	6 (14)	3 (9)		0 (0.0)	9 (9.8)	
**MUAC**						
MUAC, Mean ± SD, (millimeters)	116.73 ± 7.01	122.60 ± 10.18	0.003	113.22 ± 14.69	124.52 ± 10.09	0.003
< 110 mm, n (%)	5 (11)	5 (14)	<0.001	3 (33.3)	10 (10.6)	0.160
110mm-124mm, n (%)	34 (77)	12 (34)		4 (44.4)	38 (40.4)	
125mm-135mm, n (%)	5 (11)	15 (43)		2 (22.2)	29 (30.9)	
>135mm	0 (0)	3 (9)		0 (0.0)	17 (18.1)	

* Δ = Change during the two months of intervention

When compliance was lower (<75%), children in the RUTF group had slightly higher weight gain (p = 0.005), WAZ (p = 0.014), and WHZ (p<0.001) scores whereas the height gain (p<0.001), HAZ (p = 0.008) and MUAC (p = 0.003) scores were higher in the RUSF group.

When compliance was higher (≥75%), children in the RUSF group and RUTF group had similar weight gain whereas higher height gain in the RUSF group compared to the RUTF group (p<0.003). The MUAC scores were higher with better compliance in the RUSF group (<75%) (p = 0.003).

Side effects of RUTF and RUSF were documented weekly for both cohorts as per the subjective perceptions of the mothers, reported in **Table 5**. In the RUTF group, 54 (83.1%) participants had diarrhea as a side effect at least once. In the RUSF group, 73 (39%) participants had diarrhea, and 38 (20.3%) participants had vomited at least once. When comparing the side effects of the nutritional interventions at each follow-up, the RUSF group had a lower proportion of children with diarrhea (n = 155; 8.9%) (p<0.001) at follow-up visits than those who received RUTF (n = 167; 18.2%). However, vomiting was noted to be present only in the RUSF group at 74 follow-ups (4.2%) (p<0.001), with no vomiting reports from children who received RUTF.

**Table 5:** Side effects of nutritional intervention administration.

	RUTF group (n = 65)	RUSF group (n = 187)	P-values
**Side effects at least once**			
Diarrhea, n (%)	54 (83.1)	73 (39.0)	<0.001
Vomiting, n (%)	0 (0.0)	38 (20.3)	<0.001
Abdominal pain, n (%)	0 (0.0)	0 (0.0)	-
**Number of follow-ups**	918	1748	
**Side effects**			
Diarrhea, n (%)	167 (18.2)	155 (8.9)	<0.001
Vomiting, n (%)	0 (0.0)	74 (4.2)	<0.001
Abdominal pain, n (%)	0 (0.0)	0 (0.0)	-

Together, these data suggest that RUTF intervention showed more improvement in WHZ outcomes. In contrast, the RUSF intervention showed more improvement in average height, HAZ, and MUAC outcomes. We found that a higher compliance rate did correlate with the growth parameters. Of note, the MUAC and average height showed significantly better improvement in the high-compliance RUSF cohort compared to the RUTF cohort after the 2-month intervention.

## 4. Discussion

To our knowledge, our study was the first for the treatment of SAM globally to compare RUTF to a locally-made RUSF. This study compared two cohorts of acutely malnourished children in a rural district of Pakistan who received up to 2 months of RUTF or RUSF intervention. From these data, we assessed the improvement in growth parameters of children enrolled at nine months of age who had a WHZ < -2. This present study provides evidence of the efficacy of SAM/MAM treatment protocols, community-based management of acute malnutrition (CMAM), and different formulas for RUSF. By understanding the strengths and weaknesses of nutritional intervention strategies, we can improve the efficacy, accessibility, and affordability of nutritional supplementation for acutely malnourished children in resource-constrained settings [23].

Our trial was a quasi-experimental study; there were potential confounders in the population characteristics (e.g., WAZ and WHZ in the neonatal period), which may account for the differences in clinical profiles seen in either interventional group. Although both cohorts shared similar anthropometric profiles at the time of intervention (e.g., nine months of age), growth trajectories differed in the months preceding the intervention, as shown in **Fig 2**. Children in the RUTF group had higher WAZ and WHZ from 1 to 6 months. The RUTF group showed some regression of WAZ and WHZ between 6–9 months before the intervention, with ensuing similar anthropometric profiles at 9 months in both RUSF and RUTF groups (**Fig 2**). Both RUSF and RUTF groups improved weight and WAZ after the intervention but there was no statistical difference to the degree of improvement they caused. This could be because of the RUSF product itself or simply the phenomena of regression to the means across both groups. The trajectory of both the groups was similar regarding HAZ from 1 to 9 months. However, the RUTF group showed less improvement in height and HAZ after the intervention compared to the RUSF group. This may be because of chance, the nutritional intervention itself, or the different catch-up growth velocities across both groups.

We found a significant reduction in the SAM burden of children, as demonstrated by increased WHZ and MUAC in the treatment cohorts in both groups. Still, we found poor concordance between these two indicators in either supplementation group, agreeing with previous observations [24, 25]. We found that the degree of effectiveness for each supplementation strategy in reducing the incidence of wasting or severe wasting varied depending on the metric used, with greater improvement seen when measuring WHZ—nevertheless, a noticeable reduction in SAM burden as measured by either score. Our findings suggest that there may be no appreciable difference in efficacy against wasting between RUTF and RUSF since we found WHZ to be better responsive to RUTF and MUAC better responsive to RUSF.

RUTF is already established for use in acutely malnourished children for outpatient therapeutic programs (OTPs), whereas RUSF is used for supplementary feeding programs (SFPs) [26]. In the last two decades, there has been interest in developing locally produced ready-to-use foods, which is more acceptable due to the use of local, readily-consumed ingredients (e.g., maze in AchaMum) used in their formulations [27]. In this study, families provided with locally-made RUSF demonstrated significantly greater levels of compliance, suggesting that there is greater cultural support for locally-made nutritional supplements. Considering the logistical and financial burden upon health systems to procure imported RUTF, we expect that locally-made RUSF may be used safely in providing nutritional intervention to malnourished children in LMICs [28]. The chemical composition of the nutritional interventions is summarized in **Table 6**.

**Table 6:** Chemical composition of AchaMum and Plumpy’Nut.

Nutrition Facts Composition Per 100g Serving		
	Achamum	Plumpy’Nut
Energy, kcal	513–550	520–550
Fat, g	30–38.6	26–36
Protein, g	12.6–15.4	13–16
**Vitamin & Minerals**		
Vitamin A, mg	0.6–1.0	0.8–1.1
Thiamin, mg	0.6–1.3	≥0.5
Riboflavin, mg	0.8–2.2	≥1.6
Niacin, mg	5.3–15	≥5
Pantothenic acid, mg	2.5–4.5	≥3
Pyridoxine, mg	0.6–1.5	≥0.6
Biotin, μg	12–85	≥60
Folic acid, μg	210–254	≥200
Cobalamin, μg	1.3–2.5	≥1.6
Vitamin C, mg	53–132	≥50
Vitamin D, μg	7–23	15–20
Vitamin E, mg	16–30	≥20
Vitamin K, μg	23–38	15–30
Calcium, mg	300–545	300–600
Copper, mg	0.5–2	1.4–1.8
Iodine, μg	85–150	70–140
Iron, mg	8–13	10–14
Magnesium, mg	80–150	80–140
Phosphorus, mg	300–490	300–600
Potassium, mg	760–1,210	1,100–1,400
Selenium, μg	8–37	20–40
Sodium, mg	<290	<290
Zinc, mg	11–15	11–14

Our study has a few limitations. First, we documented the compliance within each household as per the number of sachets returned; we do not know for sure whether the target child consumed the supplement or shared it with other members of the household. This may be one of the reasons explaining the comparatively lower weight gain in the RUSF group despite higher compliance rates than the RUTF group. Second, the nutritional interventions differed by their dosing regimens. RUTF was dosed according to the child’s weight regardless of the severity of acute malnutrition, while RUSF was administered dependent upon weight in children with SAM and MAM children who received 2 sachets only irrespective of their weight. Third, side effects were self-reported by parents and not by trained medical professionals. Fourth, although both cohorts were established in the Matiari district of Pakistan, the baseline characteristics of the RUTF group were different from the RUSF group, and the studies were independently completed two years apart. Nevertheless, we observed a comparable prevalence of wasted and underweight children in both supplementation groups before the intervention. Finally, we do not know whether diarrhea was due to an underlying infectious etiology as a side effect of supplementation, as we did not collect stool samples for further workup.

## 5. Conclusion

Our paper tests the current guidelines that SAM should be treated with RUTF and MAM with RUSF. We test this convention in a real-world quasi-experimental design. Chemically, there is negligible difference in RUSF vs RUTF beyond ingredients. Here, we also found no difference in effectiveness. Our paper challenges the prevalent dogma that SAM and MAM be treated with RUSF and RUSF, respectively. We feel that they are both interchangeable and the choice should be made on availability, affordability, and practicality. Complexities are present in the management of SAM and MAM which may be unnecessary and unfounded. Our data supports that both RUSF and RUTF may be used continually for SAM and MAM treatment with the only difference being dosage.

## Supporting information

S1 Datasets (ZIP)
